# Isomer-Resolved Mobility-Mass Analysis of α-Pinene Ozonolysis Products

**DOI:** 10.1021/acs.jpca.2c03366

**Published:** 2022-07-21

**Authors:** Aurora Skyttä, Jian Gao, Runlong Cai, Mikael Ehn, Lauri R. Ahonen, Theo Kurten, Zhibin Wang, Matti P. Rissanen, Juha Kangasluoma

**Affiliations:** † Institute for Atmospheric and Earth System Research/Physics, University of Helsinki, FI-00014 Helsinki, Finland; ‡ College of Environmental and Resource Sciences, Zhejiang University, Hangzhou, 310058, China; § Department of Chemistry and Institute for Atmospheric and Earth System Research (INAR), University of Helsinki, 00014 Helsinki, Finland; ∥ Aerosol Physics Laboratory, Department of Physics, Tampere University, 33720 Tampere, Finland; ⊥ Karsa Ltd., A. I. Virtasen aukio 1, 00560 Helsinki, Finland

## Abstract

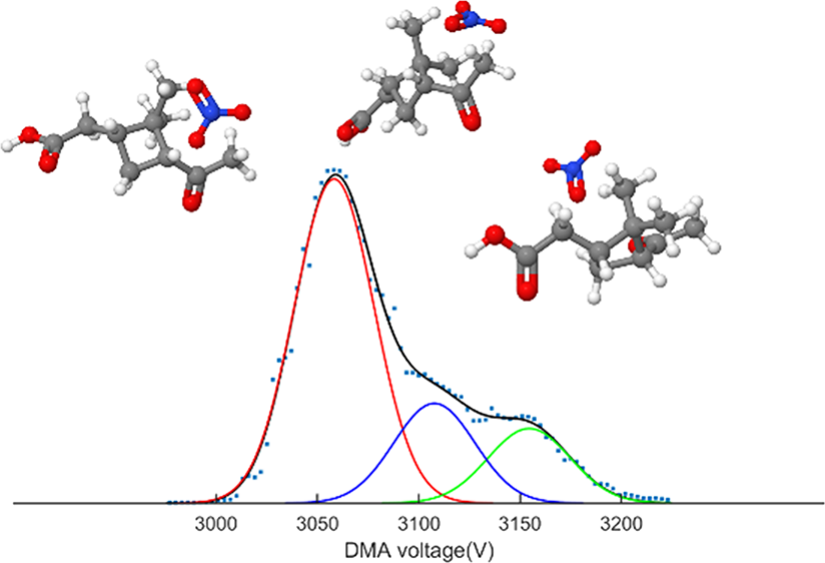

Highly oxygenated organic molecules (HOMs) are important sources of atmospheric aerosols. Resolving the molecular-level formation mechanisms of these HOMs from freshly emitted hydrocarbons improves the understanding of aerosol properties and their influence on the climate. In this study, we measure the electrical mobility and mass-to-charge ratio of α-pinene oxidation products using a secondary electrospray-differential mobility analyzer-mass spectrometer (SESI-DMA-MS). The mass-mobility spectrum of the oxidation products is measured with seven different reagent ions generated by the electrospray. We analyzed the mobility-mass spectra of the oxidation products C_9–10_H_14–18_O_2–6_. Our results show that acetate and chloride yield the highest charging efficiencies. Analysis of the mobility spectra suggests that the clusters have 1–5 isomeric structures (i.e., ion-molecule cluster structures with distinct mobilities), and the number is affected by the reagent ion. Most of the isomers are likely cluster isomers originating from binding of the reagent ion to different sites of the molecule. By comparing the number of observed isomers and measured mobilities and collision cross sections between standard pinanediol and pinonic acid to the values observed for C_10_H_18_O_2_ and C_10_H_16_O_3_ produced from oxidation of α-pinene, we confirm that pinanediol and pinonic acid are the only isomers for these elemental compositions in our experimental conditions. Our study shows that the SESI-DMA-MS produces new information from the first steps of oxidation of α-pinene.

## Introduction

Atmospheric aerosols affect the atmosphere by scattering sunlight and acting as cloud condensation nuclei and thus influence the climate. Vapors with low volatility are a major source of secondary organic aerosol (SOA). They condense onto existing particle surfaces and, in some conditions, form new particles via homogeneous nucleation.^1^ Certain volatile organic compounds (VOCs) rapidly oxidize in the atmosphere and form highly oxygenated organic molecules (HOMs) via autoxidation, becoming far less volatile.^2^ Understanding these oxidation processes is crucial for understanding atmospheric transformation and SOA formation.

One of the most important atmospheric VOC is α-pinene (C_10_H_16_). Oxidized α-pinene products contribute to both nucleation and condensational growth of SOA particles.^3^ Although the α-pinene + O_3_ and α-pinene + OH systems have been extensively studied both experimentally and computationally, the individual reaction steps leading to. e.g., HOM formation are still poorly quantified and constrained.^4−8^ One major challenge is the large number of potential reaction channels, especially for the more oxidized intermediates.

One of the experimental methods for speciating reaction products is to measure their electrical mobility and compare it to the theoretical prediction. Krechmer et al.^9^ and Zhang et al.^10^ have previously studied the electrical mobility and mass of several α-pinene oxidation products using an ion mobility spectrometer coupled to a mass spectrometer (IMS-MS). They detected the oxidation products of α-pinene C_8–10_H_12–18_O_7–13_ (monomers) and C_16–20_H_24–32_O_11–18_ (dimers). Krechmer et al.^9^ determined the number of oxidation product isomers based on the measured electrical mobilities. For almost all the examined oxidation products, only one or two isomers were observed. Zhang et al.^10^ measured multiple organic compounds, such as amines, alcohols, carbonyls, carboxylic acids, esters, phenols, and organic sulfates, with IMS-MS instrumentation. They calculated collision cross sections (CCS) for the measured compounds and showed that it is possible to distinguish between structural isomers (with the same elemental composition but different functional groups) based on the CCS. Offline methods have been also used for identifying the oxidation products of α-pinene. For example, Yu et al.^11^ used gas chromatography-MS with derivatization to identify the products.

The previous studies experimentally scoped the oxidation products for molecules C_8–10_H_12–18_O_>6_ because of the limitations in their ionization method that is not sensitive to less oxidized products (e.g., due to their lower ability to form H-bonds with the charger ions). The far more abundant less oxidized products have much larger variation in volatilities and thus a tendency to grow particles, making their speciation a more difficult, yet arguably more important task.

In this study, we demonstrate a new technique for investigating VOC oxidation. We introduce a combination of secondary electrospray ionization, differential mobility analyzer and mass spectrometer (SESI-DMA-MS) with which the electrical mobility and mass-to-charge of the products are determined. α-pinene was oxidized by ozone at approximately 1 min reaction time, and the mobility-mass spectrum for the formed oxidation products was measured with seven different reagent ions. We examine the relative ionization efficiency of the various reagent ions. From the mobility spectra, we determine the number of isomeric compounds, which can be either isomers of the neutral oxidation products or isomers of the oxidation product and reagent ion cluster, and interpret them with the help of reference species. These experiments demonstrate novel methodology for studying reagent ion clustering together with inspecting the first steps of gas-phase α-pinene oxidation.

## Methods

Figure S1 shows a schematic diagram of the experimental setups used for investigating the ozonolysis products of α-pinene, and they are briefly described below. α-Pinene was oxidized in a flow tube and using a potential aerosol mass (PAM) chamber in setups A and B, respectively. In setup C, pinanediol and pinonic acid, which are possible products of α-pinene oxidation, were evaporated to the gas phase using a heater and also by directly electrospraying them. The generated α-pinene ozonolysis products were measured using a DMA-MS in all experiments. Details of the DMA mobility and resolution calibration, peak fitting procedures and equations to calculate the reduced mobility, and collision cross sections are given in the SI. All experiments are collected to Table S1.

### Flow Tube

α-Pinene was brought to the gas phase using a bubbler with N_2_ flow of 0.1 L min^–1^. Ozone was produced using a UV light-based ozone generator (UVP Ozone Generator, Analytik Jena, US) to a flow of 0.1 L min^–1^ synthetic air. These flows are mixed to a flow of 5 L min^–1^ synthetic air and taken to a glass flow tube (length of 200 cm, diameter of 6 cm, residence time of ∼1 min). The concentration of α-pinene in the flow tube was 120 ppm, and the concentration of ozone was approximately 300 ppb. The described experimental conditions were kept constant for measurements with all the reagent ions.

The α-pinene + O_3_ reaction has a near unity OH yield,^12^ and thus oxidation by OH is nearly equally important.^13^ No OH scavengers were applied in the work. This configuration of high α-pinene concentration was chosen to maximize the yield of lower oxidized reaction products due to prompt RO_2_ + RO_2_ and RO_2_ + HO_2_ termination of the autoxidation processes. While the high α-pinene concentration ensures that no second generation oxidation initiation takes place, the first generation of RO_2_ from both the OH and O_3_ initiation and their mutual reactions can be a source of the observed C_10_H_16_O_3_. The potential reactions of primary RO_2_ from most α-pinene + oxidant combinations have been charted in recent works,^14−17^ while the subsequent generations of RO_2_ (and RO) from the self- and cross-combination reactions propagating the autoxidation chain have received far less attention.^15,17^ The detailed mechanisms potentially leading to C_10_H_16_O_3_ products are discussed later in this work.

### PAM Chamber

To investigate the possible effects of the oxidation conditions and lower α-pinene concentration on the formed oxidation products and resulting mobility spectra, similar experiments were also carried out using a potential aerosol mass (PAM) chamber (Figure S1), setup B). In the PAM chamber experiments, α-pinene was sprayed from a syringe and evaporated into a carrier gas. An N_2_ flow (0.3 L min^–1^) was used to carry α-pinene to the chamber, where ozone was produced using UV lamps. The clean air through the chamber was 10 L min^–1^. These measurements were performed with four increasing concentrations of ozone (60–650 ppb) and α-pinene (31–50 ppb) listed in Table S1. We hereafter refer to these different PAM chamber settings using the abbreviations PAM1, PAM2, PAM3, and PAM4.

**Figure 1:**
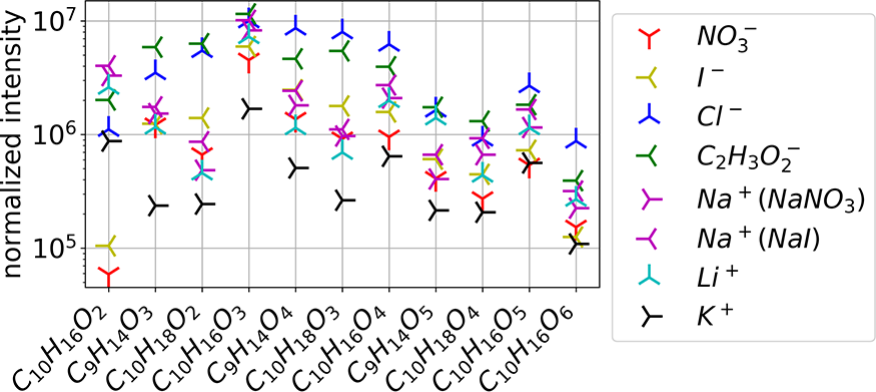
Normalized intensity of the clusters measured in the flow tube ozonolysis experiments.

### Heater

A heater was used to evaporate *cis*-pinonic acid (C_10_H_16_O_3_, Sigma-Aldrich, 98%) and (1*S*,2*S*,3*R*,5*S*)-(+)-pinanediol (C_10_H_18_O_2_, Sigma-Aldrich, 99%) from the solid phase to the gas phase. These standard compounds are expected to have only one structure. They were heated to 30–100 °C in a tube with a flow of 1 L min^–1^, which was taken to the electrospray chamber.

### Secondary Electrospray Ionization (SESI)

The neutral target molecules are charged using the SESI scheme.^18^ The electrospray needle is placed approximately 1 cm away from the DMA inlet slit, and the sample flow flows perpendicular to the ion trajectories through the electrospray chamber. Some fraction of the collisions of the reagent ions and target molecules lead to cluster formation, which is immediately drawn into the DMA against a N_2_ counter flow of 0.5 L min^–1^ through the inlet slit. Four different salts were used to generate the reagent ions: sodium nitrate (NaNO_3_), sodium iodide (NaI), lithium chloride (LiCl), and potassium acetate (CH_3_COOK). All solutions were used both in positive and negative ion mode, and the resulting reagent ions were NO_3_^–^, I^–^, Cl^–^, CH_3_COO^–^, Na^+^ (from two different liquids), Li^+^, and K^+^. Concentrations of the salts were 0.5 mM dissolved in methanol, which was observed to minimize salt cluster formation. The pressure of the electrospray solvent vial was 40–60 mbar above that of the electrospray chamber, leading to spray currents in the range of 60–100 nA. The potential difference between the needle tip and the DMA upper electrode was 2.5–3 kV. The reaction time for charging was in the range of 0.1 ms. The silica-bordered glass capillary needle was 30 cm long, with an outer diameter of 360 μm, inner diameter of 75 μm, and tip diameter of 30 μm. In the flow tube experiments, all the reagent ions were used, whereas all the PAM chamber measurements were conducted with NO_3_^–^. In the heater experiments, three reagent ions (NO_3_^–^, I^–^, and Na^+^) were applied. When pinanediol and pinonic acid were sprayed directly using standard electrospray ionization, the solution was mixed with NaNO_3_ or NaI, leading to charging with NO_3_^–^, I^–^, and Na^+^. We note that some fractions of the obtained signals may originate from extractive electrospray ionization (EESI),^19^ in which particles are extracted to the liquid droplets and molecules thereafter become ionized as in ESI. However, as the residence time for the electrospray generated ions in the ionization region is in the range of 0.1 ms (vs droplet evaporation time scales of 100 ms^20,21^) and there is no additional heating of the droplets, we speculate that there is insufficient time for particle extraction and molecule ionization through ion evaporation, and therefore most of the observed cluster ions are a result of the collisions between the reagent ions and the neutral molecules.

### Differential Mobility Analyzer (DMA)

The DMA used in this study was the DMA P5 manufactured by SEADM, Spain.^22^ The DMA was operated in a counter flow mode such that N_2_ was fed to the closed sheath air flow circulation, and an excess of 0.5 L min^–1^ was flowing out of the DMA inlet. The classified ions were taken to an electrometer and a mass spectrometer sampling in parallel downstream of the DMA.^23^

### Mass Spectrometer

Mass-to-charge-ratios of the clusters classified using the DMA were measured using an atmospheric pressure time-of-flight mass spectrometer (APi-TOF).^24^ The mass resolution of the APi-TOF in our experiments was around 5000, which is sufficient to resolve the elemental composition of the generated oxidation products.

### Ozonolysis of α-Pinene

α-Pinene ozonolysis is among the most studied tropospheric oxidation processes and is responsible for a significant fraction of atmospheric SOA (e.g., refs. (6,15,16,25 and 26)). In the atmosphere, α-pinene can be oxidized by ozone, hydroxyl radicals, or nitrate radicals.^27^ In our experiments, oxidation was initiated by a reaction of α-pinene with ozone, yet oxidation initiation by OH is nearly as important due to co-produced OH. α-Pinene can react with ozone and OH through different reaction channels, which have been described in detail in several publications.^7,28^ Typical oxidized products include C_8_H_12–14_O_3–4_, C_9_H_14–16_O_2–4_, and C_10_H_14–16_O_2–4_. In addition, α-pinene can gain high oxygen numbers in a short time via autoxidation in a sequence of intramolecular hydrogen shifts and subsequent oxygen addition reactions.^4,29^ The ozonolysis products expected to form through autoxidation are C_8–10_H_12–18_O_4–12_ (monomers) and C_16–20_H_24–34_O_8–16_ (dimers). In addition to these, oxidation products may fragment, and therefore also products with a shorter carbon skeleton are formed.^8^ This fragmentation can occur in different positions of the carbon skeleton forming two fragments with, for example, 1 and 9, 2 and 8, or 3 and 7 carbon atoms.

Of special interest for our current study are the products with compositions C_10_H_18_O_2_ and C_10_H_16_O_3_, commonly associated with pinanediol and pinonic acid, respectively. Yet, also some others of the numerous oxygenated radicals and closed-shell reaction products deriving from α-pinene oxidation could in principle yield these observed signals. While pinanediol is apparently the sole previously reported product with a composition of C_10_H_18_O_2_, the C_10_H_16_O_3_ formula has been associated with various compositions, most notably with pinonic acid formed through a dioxirane in the ester channel (see Figure 7 from Winterhalter et al.^38^). Other proposed C_10_H_16_O_3_ structures include, e.g., a bicyclic dihydroxycarbonyl product^12^ and a hydroperoxycarbonyl that has lost the α-pinene cyclobutyl ring^15^ (see Table 1 below). However, these products result from complex formation pathways (e.g., the dihydroxycarbonyl product^12^ would require two bimolecular RO_2_ reaction steps), making their formation in the current experiments less likely, though not strictly impossible.

In the manuscript, we discuss two types of isomeric compounds: isomers of the neutral oxidation product and isomers of the neutral oxidation product clustered with the reagent ion. We call these “molecule isomers” and “cluster isomers”, respectively, if they can be separated, and “isomers” otherwise.

**Table 1:**
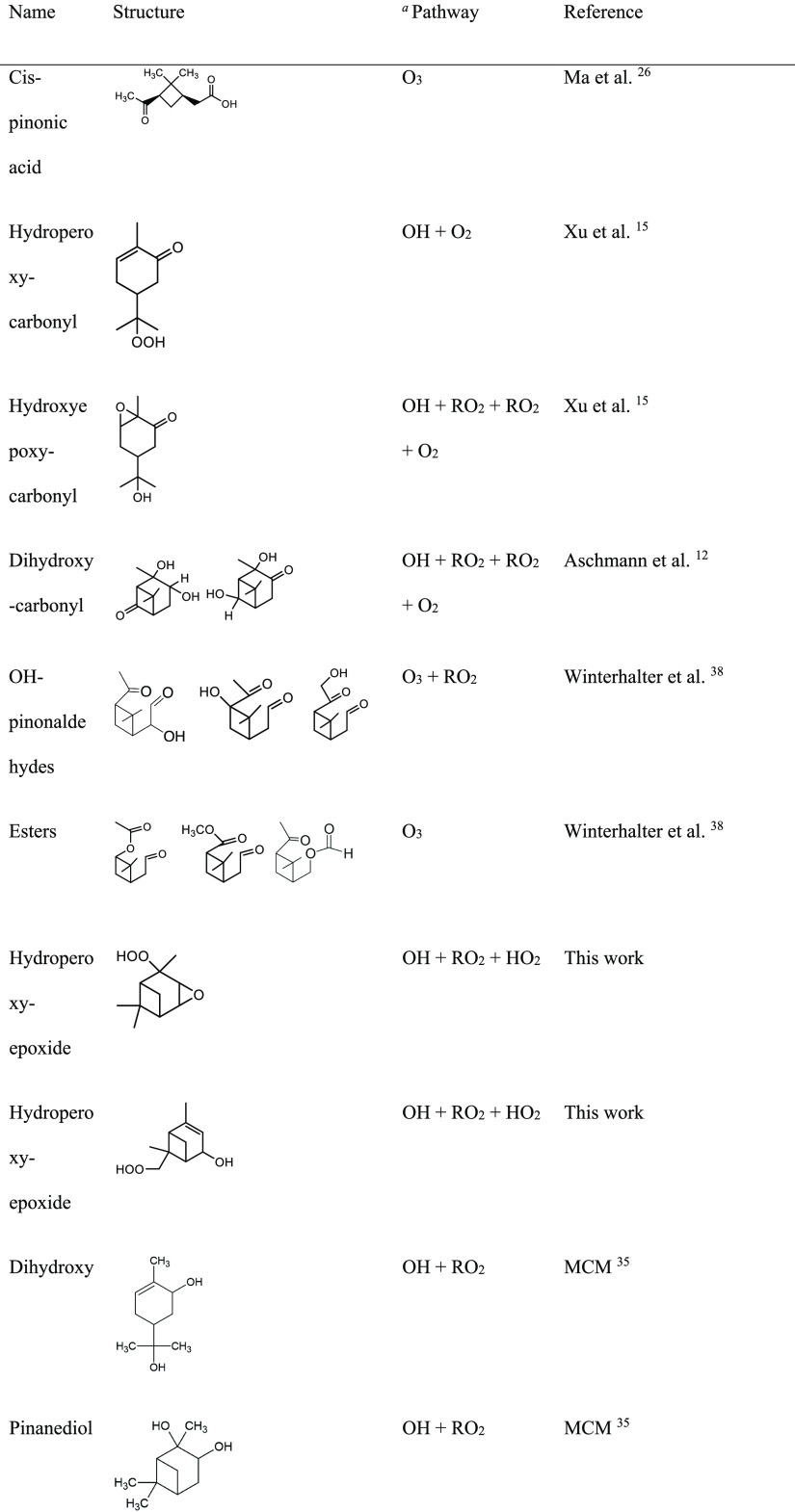
Proposed Primary α-Pinene Oxidation Products with the Elemental Composition C_10_H_16_O_3_

a O_2_ addition steps have been omitted in the description.

## Results

### Detected Oxidation Products

α-Pinene ozonolysis product compositions observed in the flow tube experiments are listed in Table S2, and the corresponding mass spectra are presented in Figures S4–S6. It is good to note that the charging process may induce transformations in the detected elemental compositions, and we detect the result spectra from oxidation and charging chemistry (see, e.g., reference (30)). Similar to Zhao et al.,^31^ with positive reagent ions, both monomers and dimers are clearly detected, while with negative reagent ions, only monomers are detected in our experimental conditions. As shown in Table S2, the monomer products listed by Ma et al.^7^ (C_8_H_12_O_3–4_, C_9_H_14_O_2–4_, and C_10_H_16_O_2–4_) were observed. We did not detect the α-pinene HOM with oxygen numbers O > 8 reported in several other studies (see, e.g., ref (4) and references therein), as expected due to the chosen experimental design. There could be several other reasons for this too: In typical chemical ionization (CI) methods, the reaction time for clustering is about 2–3 orders of magnitude longer than in our SESI (i.e., 0.1 vs 300 ms). Another major difference here is the near absence of neutral nitric acid HNO_3_ in our experiments. The presence of neutral HNO_3_ leads to main reagent ion being (HNO_3_)NO_3_^–^ in the previous studies,^32^ which is selective for higher oxidation products, whereas NO_3_^–^ that we used clusters easily with virtually all oxidation products. Therefore, both the difference in the charging scheme and in the experimental conditions favor the detection of the lower oxidized products. The fact that we mostly detect oxidation products with low oxidation states, O_3_ showing the highest signal, suggests that the main ionization mechanism is SESI instead of EESI, as similar results have been recently reported.^33^

In addition to these early-stage oxidation products, compounds with less than 10 carbon atoms were detected. C_7_H_10_O_4_, C_7_H_12_O_3–5_, C_8_H_14_O_3–7_, C_9_H_14_O_5–6_, and C_9_H_16_O_4–5_ were observed and are formed because of fragmentation of the α-pinene carbon skeleton. C_8_H_14_O_*x*_ fragments with higher oxygen numbers are detected with negative reagent ions (x = 3–7) compared to positive reagent ions (x = 3–5).

There are some differences also when comparing mass spectra between negative reagent ions. For compounds C_8_H_14_O_*x*_, only C_8_H_14_O_3–5_ were detected using C_2_H_3_O_2_^–^. In contrast, C_8_H_14_O_3–7_ were detected with other negative reagent ions. Also, the compounds C_10_H_14_O_*x*_ were detected with *x* up to 3 using C_2_H_3_O_2_^–^ but with other negative ions, *x* was up to 5 (with I^–^ and Cl^–^) or 6 (with NO_3_^–^). For the mass spectra measured using positive reagent ions, we detected C_10_H_14_O with Li^+^ and K^+^ but not with Na^+^. However, most of the compounds were detected with all reagent ions, especially monomers.

For dimer compounds, there are more differences between the reagent ions. With Na^+^ from NaI as the reagent ion, different compounds are detected than with other positive reagent ions. This is unexpected because both Na^+^ ions should be the same ions and behave similarly regardless of from which salt the ion is generated. This reagent ion salt produces also dimers and trimers, but their concentration was so low that they are unlikely to contribute to the charging because we did not observe clusters charged with, e.g., Na_2_I^+^. It is possible that some fraction of the charging takes place in the liquid phase droplets if some of the neutral molecules solvate before the coulomb explosions. Alternatively, differences in the Na^+^ concentration may explain some observed differences. However, we cannot unambiguously explain why different molecules are detected when Na^+^ is formed by two different precursor compounds. Yet, as shown below in Table 3, the results for the observed number of isomeric species are very similar with both sources of Na^+^.

The most significant difference among the spectra obtained with different charging ions was the charging efficiency, which is observed as a difference in the detected signal intensities. To take into account the reagent ion concentration, we normalized the observed cluster signals by dividing the signal intensity with the intensity of the reagent ion measured by the electrometer. Figure 1 shows the reagent ion-normalized intensities of the oxidation products exhibiting the highest signal intensities collected from the flow tube experiments, where the measurement conditions were constant for all reagent ions. We can conclude that the charging efficiency was generally the highest when using Cl^–^ and C_2_H_3_O_2_^–^ and the weakest when using NO_3_^–^, Li^+^, and K^+^. The differences in charging efficiency between negative and positive charging ions were not significant, suggesting probably that other chemical characteristics of the ions such as size and shape (which determine, for example, the ability to form H bonds with the analyte molecules) are affecting the charging efficiency more than polarity.

### Number of Isomers

**Figure 2:**
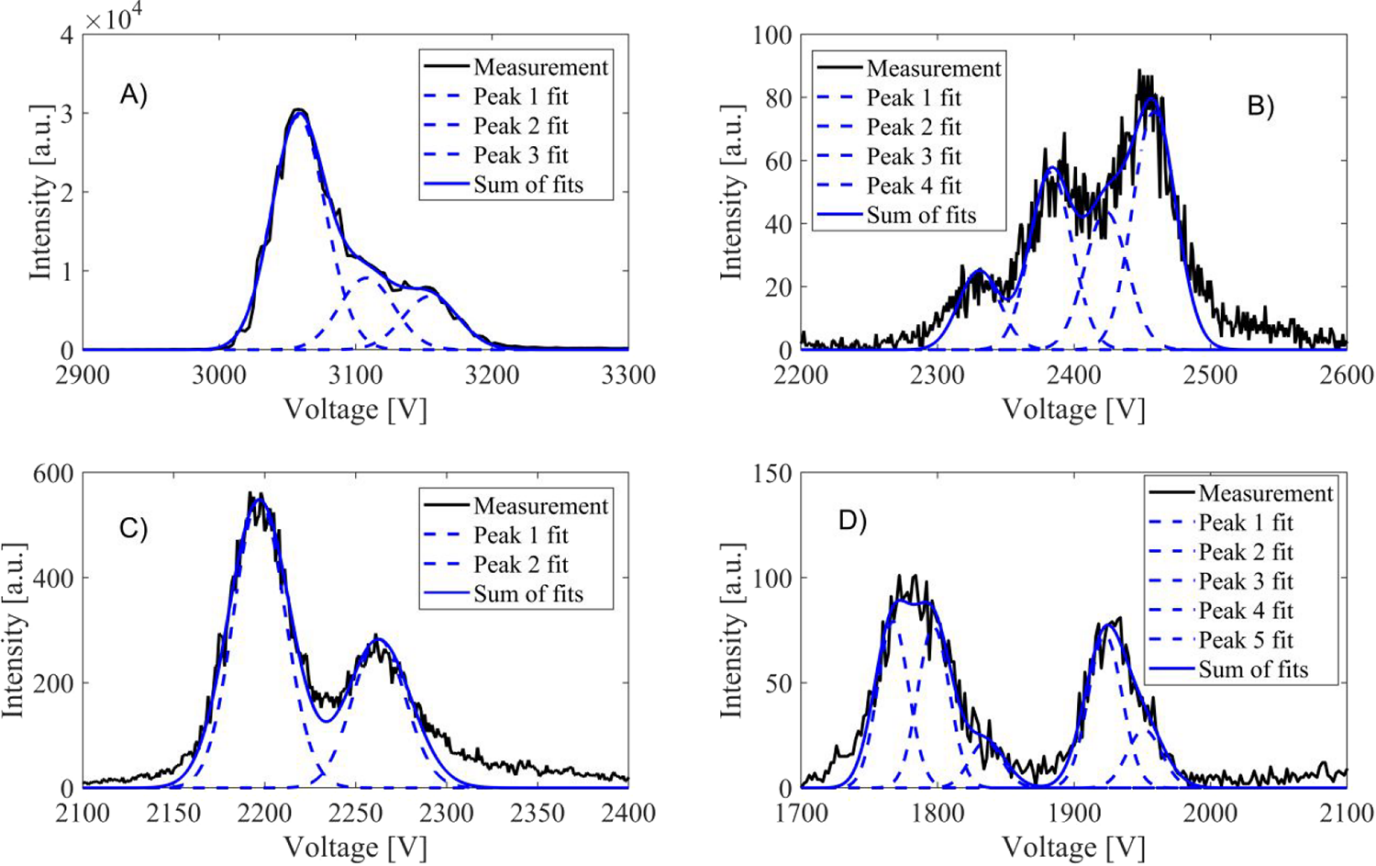
Measured mobility spectrum for (A) C_10_H_16_O_3_NO_3_^–^, (B) C_10_H_16_O_4_Na^+^, (C) C_10_H_16_O_2_K^+^, and (D) C_10_H_16_O_4_Cl^–^. (A) is from PAM1 experiments while others are from flow tube experiments.

In this section, we discuss the number of isomers of the generated clusters from different measurements, especially in the context of reaction pathways leading to the formation of C_10_H_16_O_3_ products. In cases where it is difficult to decide the number of fitted peaks, a minus or plus sign indicates if also one peak more (+) or less (−) might be a reasonable number of isomers for a certain cluster (see the SI for more discussion and Figure S3). Figure 2 presents example mobility spectra for four different compounds. The mobility spectra clearly demonstrate the presence of isomeric compounds that are measured for the same elemental composition.

### Pinonic Acid and Pinanediol

Ozonolysis of α-pinene produces four primary RO_2_ with a C_10_H_15_O_4_ composition,^6^ while OH addition produces two distinct C_10_H_17_O_3_, and OH abstraction produces several C_10_H_15_O_2_ peroxy radicals.^15,34^ The OH abstraction pathways have been commonly neglected, (e.g., also in MCM^35^) having only a 12% yield,^34^ with the allylic abstraction from the 6-membered ring accounting for about 3/4. This pathway could also yield C_10_H_16_O_3_, if the primary C_10_H_15_O_2_ radical reacts with another RO_2_ producing an alkoxy radical that isomerizes by H shift (or epoxidation) to an alkyl radical, adds an O_2_, and terminates to a hydroperoxide in a RO_2_ + HO_2_ reaction. Two such options are sketched in Scheme S1. The OH addition and the subsequent sole ring-opened RO_2_ have generally been considered the route to HOM by OH in this system.^15−17^ Xu et al.^15^ found two distinct C_10_H_16_O_3_ products from this pathway; a hydroperoxycarbonyl product through 1,6-H shift from the carbon having the OH followed by a carbonyl formation by an O_2_ reaction (see Schemes 3 and 4 from their work). This was the 2nd fastest unimolecular reaction for the ring-opened *anti*-RO_2_ product, and the third fastest when considering both stereoisomers. Yet, another cyclic C_10_H_16_O_3_ product with an epoxide, a carbonyl, and a hydroxy group was found from the subsequent RO_2_ chemistry (Scheme 3 from Xu et al.^15^ and Table 1), but formation of this product seems less likely due to the more complex formation mechanism. Nevertheless, the balance between these product pathways is ultimately determined by the temperature and the concentration of the co-reagents NO, RO_2_, and HO_2_.

Also, the four ozonolysis-derived C_10_H_15_O_4_ radicals can yield C_10_H_16_O_3_ hydroxy pinonaldehyde products by the well-known Russell mechanism,^36^ which occurs through a tetroxide intermediate and forms an alcohol with one more H atom and a carbonyl with one less H atom than the precursor peroxy radicals. Also the C_10_H_18_O_2_ could be produced similarly from the C_10_H_17_O_3_ ring-opened OH addition product, leading to a tertiary dihydroxy compound (see Table 1).

While there remains a multitude of pathways to potential C_10_H_16_O_3_ products in the α-pinene + O_3_/OH system, pinonic acid is the most known of the C_10_H_16_O_3_ molecule isomers and according to current measurements the most likely origin for the measured signal (see below). That is, only one molecule isomer appears to be detected in the experimental work. Several molecule isomeric C_10_H_16_O_3_ products could be formed also through subsequent oxidation of the primary reaction products but are excluded by the experimental design; the very high excess of α-pinene prevents second oxidation initiation in this system. Different potential C_10_H_16_O_3_ products and their possible formation routes are summarized in Table 1.

Table 2 shows the number of isomers derived from peak fitting to the mobility spectra from the evaporation and electrospray experiments, where standard pinonic acid and pinanediol were measured, together with the number of isomers for C_10_H_16_O_3_ and C_10_H_18_O_2_ clusters from flow tube and PAM experiments. The table shows that the cluster of C_10_H_16_O_3_ or C_10_H_18_O_2_ with a reagent ion has two or three isomers. In PAM3 and PAM4 experiments, four isomers are observed for C_10_H_16_O_3_NO_3_^–^. We assume pinonic acid and pinanediol detected in the evaporation and electrospray experiments have only one molecule isomer as they are produced from standard samples. Therefore, it can be inferred that the peaks in the mobility spectrum originate from cluster isomers (not molecular isomers of the oxidation product). The numbers in the table also suggest that in ESI experiments, less cluster isomers are formed than in the evaporation experiments. This may be explained by that in ESI, only stable clusters form in the liquid phase, while in SESI charging of the oxidation products, some more unstable clusters in addition are detected. Another possibility, which we only speculate, is that pinonic acid and pinanediol isomerize when heated and therefore also one molecular isomer is detected in the evaporation experiments.

**Table 2:** Number of Isomers for Pinonic Acid and Pinanediol Charged with NO_3_^–^, I^–^, and Na^+^ from Evaporation, ESI, Flow Tube, and PAM Experiments^a^

compound	experiment	NO_3_^–^	I^–^	Na^+^
C_10_H_18_O_2_	ESI	2+	2	3–
	heater	3-	3	3+
	flow tube	2	2	2
	PAM1	3+	NA	NA
	PAM2	2+	NA	NA
	PAM3	3	NA	NA
	PAM4	3	NA	NA
C_10_H_16_O_3_	ESI	2+	3–	3–
	heater	3–	2+	3–
	flow tube	3	2	2
	PAM1	3	NA	NA
	PAM2	3	NA	NA
	PAM3	4–	NA	NA
	PAM4	4+	NA	NA

a For example, 2+ in the table means likely 2 but possibly 3 isomers. NA = not applicable.

The C_10_H_18_O_2_NO_3_^–^ cluster in evaporation experiments has three isomers, while from flow tube and PAM experiments, the number of isomers is two or three. The number of observed isomers is not larger in oxidation experiments than in evaporation experiments, which indicates that the oxidation product molecule C_10_H_18_O_2_ has only one molecular isomer. C_10_H_18_O_2_ charged with either I^–^ or Na^+^ has three isomers in evaporation experiments and two in oxidation experiments. These show that the C_10_H_18_O_2_ molecule formed in oxidation experiments has only one molecule isomer that is pinanediol.

In evaporation experiments, C_10_H_16_O_3_NO_3_^–^ showed up to three isomers. In flow tube experiments, the same cluster showed three isomers, and in PAM experiments, there are three or four. Assuming the two or three isomers in the evaporation experiment to be cluster isomers, the molecule C_10_H_16_O_3_ has only one molecule isomer, or potentially two isomers if comparing to the results from PAM3 and PAM4 measurements. Nevertheless, because in PAM3 and PAM4 measurements, the oxidation conditions were different to the flow tube measurements, they are not necessarily comparable to other measurements as explained later in the analysis of Table 3.

**Table 3:** Number of Isomers of Oxidation Products of α-Pinene from Flow Tube Charged with Different Reagent Ions^a^

compound	NO_3_^–^	I^–^	Cl^–^	C_2_H_3_O_2_^–^	Na^+^	Na^+^	Li^+^	K^+^
C_10_H_16_O_2_	4+	3+	3	4	2-	2-	2	3
C_9_H_14_O_3_	2	1	2	3-	2	2	2	2
C_10_H_18_O_2_	2	2	2	2	2	2	2+	4
C_10_H_16_O_3_	3	2	1+	3	2	2	2	–2
C_9_H_14_O_4_	3	2+	3	3	3	3	2+	2
C_10_H_18_O_3_	2	3	2	3-	3-	2	2	2
C_10_H_16_O_4_	3	3	5+	4+	5	5	4	3
C_9_H_14_O_5_	3	3	1+	4	3	3	4	2
C_10_H_18_O_4_	3	2	1	2	3-	2-	3	3
C_10_H_16_O_5_	3	3	5	4	3+	3+	3	4+
C_10_H_16_O_6_	3	4	4+	4	4	4	4	4+

a From left to right the first Na^+^ ion is from salt NaNO_3_ and the second one from salt NaI.

When C_10_H_16_O_3_ is clustered with I^–^, the cluster showed two isomers in evaporation experiments and two also in flow tube experiments. Thus, when C_10_H_16_O_3_ is clustered with I^–^, the results suggest only one molecule isomer for C_10_H_16_O_3_. When charged with Na^+^, the C_10_H_16_O_3_ cluster had three or maybe two isomers in evaporation experiments and two isomers in flow tube experiments. This also indicates that C_10_H_16_O_3_ has only one molecule isomer. Based on the analysis with all three reagent ions, it can be inferred that in our experimental conditions α-pinene ozonolysis, we can detect only one molecule isomer of the C_10_H_16_O_3_ molecule, which is *cis*-pinonic acid.

This is consistent with the conventional understanding of α-pinene oxidation, which is known to form *cis*-pinonic acid. (The formation of *trans*-pinonic acid is impossible as the two substituents on the 4-carbon ring in *cis*-pinonic acid were connected to each other in the 6-carbon ring of the α-pinene parent.) For example, Yu et al.^11^ identified only pinonic acid for the composition of C_10_H_16_O_3_ with an offline method including sample collection and derivatization, compared to our online method without a need for sample preparation. In the lack of authentic standards, we and Yu et al. used retention time/electrical mobility information of similar surrogate molecules in order to quantify the measured signals and by this were able to assign the measured signals to certain expected products. The result also indicates that none of the other speculative pathways presented in Table 1 are competitive in the experimental and ionization conditions applied here.

### Other Oxidation Products

The number of observed isomers is analyzed for C_9_H_14_O_3–5_, C_10_H_16_O_2–6_, and C_10_H_18_O_2–4_ for the flow tube experiments. Table 3 shows that each cluster has a different number of isomers depending on the reagent ion, and there are no signs showing that clusters charged with some certain reagent ion would have an especially high or low number of isomers. Most of the clusters have two or three isomers, and a slight trend for increasing number of isomers with higher oxygen numbers. Different reagent ions with the same polarity are generally likely to find a similar binding geometry with a given molecular target. This is observed especially for the chemically simple positive reagent ions (Na+, Li+, K+): the number of observed isomers (for a certain elemental composition) is similar for all positive reagents. However, the larger size and the more complex structures of some of the negative reagent ions may allow for both stronger binding and potentially more isomeric binding sites (i.e., more cluster isomers). Greater chemical complexity may also result in the different negative reagent ions showing larger differences in the number of observed isomers for a certain target molecule.

In the PAM chamber experiments (Table S3), the number of isomers for some compounds is increasing when the α-pinene and ozone concentrations are increased. This may be due to higher precursor concentrations and additional photochemistry of the reaction products forming a larger number of oxidation products with concentrations above the detection limit. However, the numbers of isomers vary mainly between three and four and only for some compounds, so generally the number of isomers is quite constant within the range of these experimental conditions. The number of observed isomers from the PAM experiments agrees well with the flow tube experiments.

More research should be done to exclude the cluster isomers. In light of the current results, the reagent ions NO_3_^–^, I^–^, and Na^+^ can bind to two or three different sites of C_10_H_16_O_3_ and C_10_H_18_O_2_ molecules. However, the number of potential clustering options can change depending on the molecule and is in general likely to increase with the size and complexity of the target molecule. We further note that our method may miss some isomeric species with low yields that will be below the detection limits or behind the main mobility peaks, as evidenced by, e.g., the pinonic acid signals from the PAM experiments. Therefore, definitive conclusions on the number of molecular isomers for other compounds are hard to make. However, it seems unlikely that the number of cluster isomers for the more highly oxidized compounds would be much less than for C_10_H_16_O_3_ and C_10_H_18_O_2_. It could thus be speculated that, e.g., the C_10_H_16_O_5_ and C_10_H_16_O_6_ compounds in Table 3 are unlikely to have more than two or at most three molecular isomers – and quite possibly have only one.

### Collision Cross Section and Inverse Reduced Mobility

The presented reduced inverse mobilities are calculated from the average DMA voltage for each mobility peak and the different isomers of the clusters are not separated. Also, peak voltages of clusters C_9_H_14_O_*x*_ and C_10_H_18_O_*x*_ are so close to each other that they are not separated here. Systematic uncertainty between experiments of the calculated inverse reduced mobility is estimated to be ±0.02 Vs cm^–2^ and for CCS ±2 Å^2^, which are based on observed variations in the mobilities and CCSs from multiple experiments that are calibrated using the THA+ ion.

Table S4 shows the reduced inverse mobility and CCS values for pinonic acid and pinanediol from the evaporation and electrospray experiments charged with NO_3_^–^, I^–^, and Na^+^. For comparison, the values of those clusters from flow tube and PAM experiments are listed in the same table (in PAM experiments, only reagent ion NO_3_^–^ was used). Both reduced inverse mobility and CCS are very similar for the clusters, which contain the oxidation product produced through ozonolysis (flow tube and PAM) and for synthetically produced oxidation products (ESI and heater). The discrepancy for pinanediol is slightly larger but may be because the mobility spectra had to be separated from C_9_H_14_O_4_ in the flow tube experiments, which increases the uncertainty in the determined 1/*Z*_0_ and CCS. However, overall, the table strongly supports the conclusion already drawn from the number of isomers in the previous section that the C_10_H_16_O_3_ and C_10_H_18_O_2_ detected in ozonolysis experiments are pinonic acid and pinanediol.

**Figure 3:**
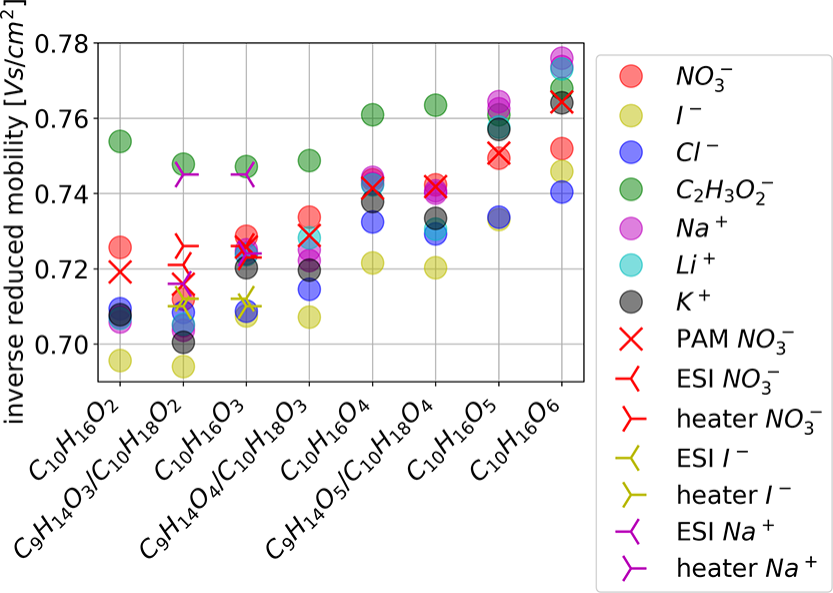
Inverse reduced mobility values for the oxidation products of α-pinene clustered with different reagent ions. Systematic uncertainty between experiments of the calculated inverse reduced mobility is estimated to be ±0.02 V s cm^–2^.

**Figure 4:**
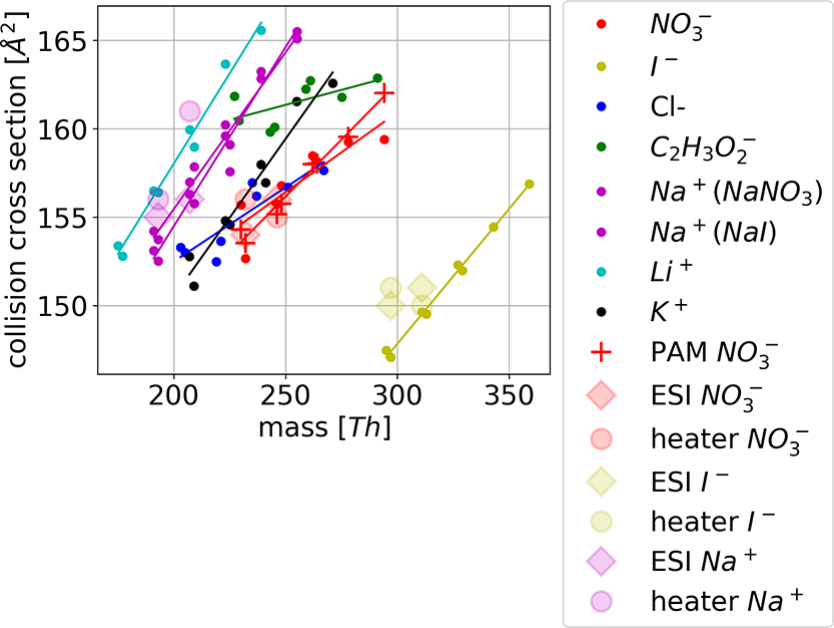
CCS for the analyzed oxidation products of α-pinene clustered with different reagent ions.

For the other oxidation products, clustering with different reagent ions yields clearly different 1/*Z*_0_ and CCS for the same neutral molecule (Figures 3 and 4 and Table S5 and S6). Further, the order of the 1/*Z*_0_ with different reagent ions is not preserved but can vary from molecule to molecule. For example, the clusters charged with C_2_H_3_O_2_^–^ have the highest 1/*Z*_0_ for products with O < 5, but for products with O = 5 or 6, the clusters charged with Na^+^ and Li^+^ have the highest 1/*Z*_0_ values. The largest 1/*Z*_0_ for clusters charged with C_2_H_3_O_2_^–^ is expected as it is the largest reagent ion. On the other hand, in most cases, the smallest 1/*Z*_0_ values are obtained for clusters charged with I^–^. Generally, clusters produced with the flow tube and PAM chamber show similar inverse mobilities. Also, mobilities of the products charged with NO_3_^–^ and I^–^ from different sources agree relatively well, but there are some discrepancies in the mobilities for products charged with Na^+^.

CCS as a function of the cluster mass shows three distinct behaviors. Li^+^, Na^+^, and K^+^ show a similar slope in the increase of the CCS with mass. The slope with C_2_H_3_O_2_^–^, NO_3_^–^, and Cl^–^ is slightly smaller than with the positive reagent ions. The smaller slope indicates that the cluster structure changes when the reagent ion binds to it: initially larger neutral molecules exhibit similar mobility to the smaller ones after the reagent ion has clustered with it. This is likely due to hydrogen bonding that may make the structure more compact. The steeper slope, on the other hand, suggests that the reagent ion binds somewhere on the outer edge of the neutral molecule, and always increases the CCS the same amount. Products charged with I^–^ are clearly heavier than the others, but also the cross section is clearly smaller. The slope is similar to the positive clusters, which are also charged by clustering of a single atom.

## Conclusions

We have shown that DMA-SESI-MS can provide new knowledge on the molecular structure and properties of atmospheric compounds and is a promising experimental tool for investigating, e.g., oxidation mechanisms and chemical ionization. To validate this technique and explore the influencing factors on the DMA-SESI-MS spectra, we performed α-pinene ozonolysis experiments and measured the oxidation products. After charging by seven different reagent ions, the mobility of the formed clusters was measured with the DMA, and the elemental composition of the oxidation product was determined using a mass spectrometer. Acetate and chloride exhibited the highest overall charging efficiency while potassium showed the lowest.

The number of isomers for a given elemental composition of an oxidation product formed in the ozonolysis of α-pinene was estimated by peak fitting to the measured mobility spectra. The number of observed isomers ranged from 1 to 5. We found that C_10_H_18_O_2_ and C_10_H_16_O_3_ formed in the oxidation of α-pinene most likely have only one structure even if they exhibit 2 or 3 peaks in the mobility spectra, as these correspond to cluster isomers, not isomers of the neutral molecule. This conclusion is based both on the measured mobilities and observed number of isomers in comparison to pinanediol and pinonic acid produced from standard stock powders. This result is consistent with Krechmer et al.^9^ where almost all analyzed oxidation products of α-pinene had only one or two isomers, although the analyzed oxidation products in that study had a higher number of oxygen atoms, >7, than in this study. Because the instrument used in this study detects also the cluster isomers, the number of molecular isomers for other measured oxidation products remains inconclusive, although it is likely 2 or less. Our results, however, demonstrate that different reagent ions bind differently to the oxidation products: acetate, nitrate, and chloride likely deform the oxidation product, while the other atomic reagent ions bind to the molecule without deforming it.

There are several ways on how the results of our study could be improved or continued. Better mobility separation can possibly be achieved by optimization of the DMA, or use of the IMS configuration, which gives better peak identification from the mobility spectrum. The electrospray ionization scheme could be improved to obtain lower detection limits for the possibly undetected isomers. For example Amo-Gonzalez et al.^39^ included a droplet desolvation configuration in the SESI. It may be possible to use more complex reagent ions that have an increased tendency to bind into a certain side or site of the molecule, providing molecule isomer separation already in the ionization stage. Further separation of the isomers may be achieved by doping the DMA sheath flow with selected vapors, as demonstrated for example for explosives.^40^ Finally, fragmentation-based isomer identification could be possible with similar methods as demonstrated elsewhere.^41^

## Supporting Information Available

Equations for reduced mobility, collision cross section and DMA calibration; Figure S1: experimental setup; Figure S2: DMA resolution function; Figure S3: peak fitting examples; Figures S4–6: measured mass spectra; Scheme S1: formation routes for C_10_H_16_O_3_; Table S1: list of experiments; Table S2: identified compounds; Table S3: number of isomers in PAM experiments; Table S4: inverse reduced mobilities of pinonic acid and pinanediol; Tables S5 and S6: inverse reduced mobilities and collision cross sections for the oxidation products in flow tube experiments (PDF)
