# Supplementary material for: Isomer-Resolved Mobility-Mass Analysis of α-Pinene Ozonolysis Products
Source: J Phys Chem A. 2022 Jul 21;126(30):5040–9. doi: 10.1021/acs.jpca.2c03366 (PMC9358649; doi:10.1021/acs.jpca.2c03366)
Supplement: Supplementary file 1 — jp2c03366_si_001.pdf [file jp2c03366_si_001.pdf]

Supplementary information for

Isomer-Resolved Mobility-Mass Analysis of α-Pinene Ozonolysis Products

Aurora Skyttä[1], Jian Gao[2], Runlong Cai[1], Mikael Ehn[1], Lauri Ahonen[1], Theo Kurten[3], Zhibin Wang[2], Matti Rissanen[4], Juha Kangasluoma[1,5,*]

[1] Institute for Atmospheric and Earth System Research/Physics, University of Helsinki, FI-00014 Helsinki, Finland

[2] College of Environmental and Resource Sciences, Zhejiang University, Hangzhou, 310058, China

[3] Department of Chemistry and Institute for Atmospheric and Earth System Research (INAR), University of Helsinki, 00014 Helsinki, Finland

[4] Aerosol Physics Laboratory, Department of Physics, Tampere University, 33720 Tampere, Finland

[5] Karsa Ltd., A. I. Virtasen aukio 1, 00560 Helsinki, Finland

Reduced mobility and collision cross section. To normalize the effect of laboratory temperature and pressure to the measured mobility, we report the reduced mobility using Eq. 1 (Gabelica et al. 2019):

$$Z_0 = Z\frac{p\,T_0}{p_0\,T} \qquad \text{(S1)}$$

where $_Z$ is the electrical mobility, p_0 is standard pressure, T_0 is standard temperature, p is pressure in the DMA and T is temperature in the DMA. In the study, we use value of p_0 = 1000 mbar for the standard pressure and value of T_0 = 273.15 K for the standard temperature. Pressure and temperature in the DMA are estimated to be p = 1013 mbar and T = 298.15 K, respectively.

The collision cross section Ω (CCS) describes the effective area transporting energy between sheath gas molecule and ion:

$$\Omega = \frac{3}{16}\left(\frac{2\pi}{\mu kT}\right)\left(\frac{zc}{N_0 Z_0}\right) \qquad \text{(S2)}$$

where μ is the reduced mass, k is the Boltzmann constant, T is the temperature in the DMA, z is the number of elementary charges of the cluster, c is elementary charge, N_0 is Loschmidt's constant and Z_0 is the reduced mobility. The reduced mass is defined as $\mu = (m_i * m_g)/(m_i + m_g)$, where m_i is mass of the charged cluster and m_g is mass of the sheath gas molecule. In this study, nitrogen (N_2) was used at the sheath gas.

DMA mobility calibration. The DMA was calibrated using tetraheptylammonium bromide (THAB, $C_{28}H_{60}NBr$) with known electrical mobility. The inverse electrical mobility (*1/Z*) for THAB monomer and dimer in the positive polarity at 293 K are 1.030 and 1.529 Vs/cm^2, respectively (Ude and Fernandez de la Mora 2005). DMA voltage is proportional to the inverse mobility. Therefore, the inverse mobility corresponding to voltage V_{DMA} can be solved as

$$Z^{-1} = \frac{V_{DMA}}{Z_{THABmon} * V_{THABmon}} \qquad \text{(S3)}$$

Where V_{DMA} is the DMA set point voltage and $V_{THABmon}$ is the measured tetraheptylammonium ion peak voltage.

DMA resolution. For mobility peak fitting, the mobility resolution of the DMA needs to be known. Resolution is defined as the mean ion mobility divided by the full width at half maximum. Practically, the DMA resolution as a function of DMA voltage was determined using the measured peaks with the highest observed resolution. The peaks with the highest resolution are the ones measured with the maximum resolution of

the instrument, as shown in Figure 3.2. The clusters with the highest resolution are usually the negatively charged reagent ion clusters, such as $(NaNO_3)_xNO_3^-$, $(NaI)_xI^-$ $(LiCl)_xCl^-$ and $(C_2H_3O_2K)_xC_2H_3O_2^-$. The resolution function of the DMA is fitted to the peaks with the highest resolution. If the resolution of a mobility peak is approximately equal to the instrument resolution, it is likely that this mobility peak consists of one compound which has only one structure. If a peak exhibits a significantly lower resolution than the instrument resolution, it may consist of multiple overlapping mobility peaks to which peak fitting is done using the fitted resolution function.

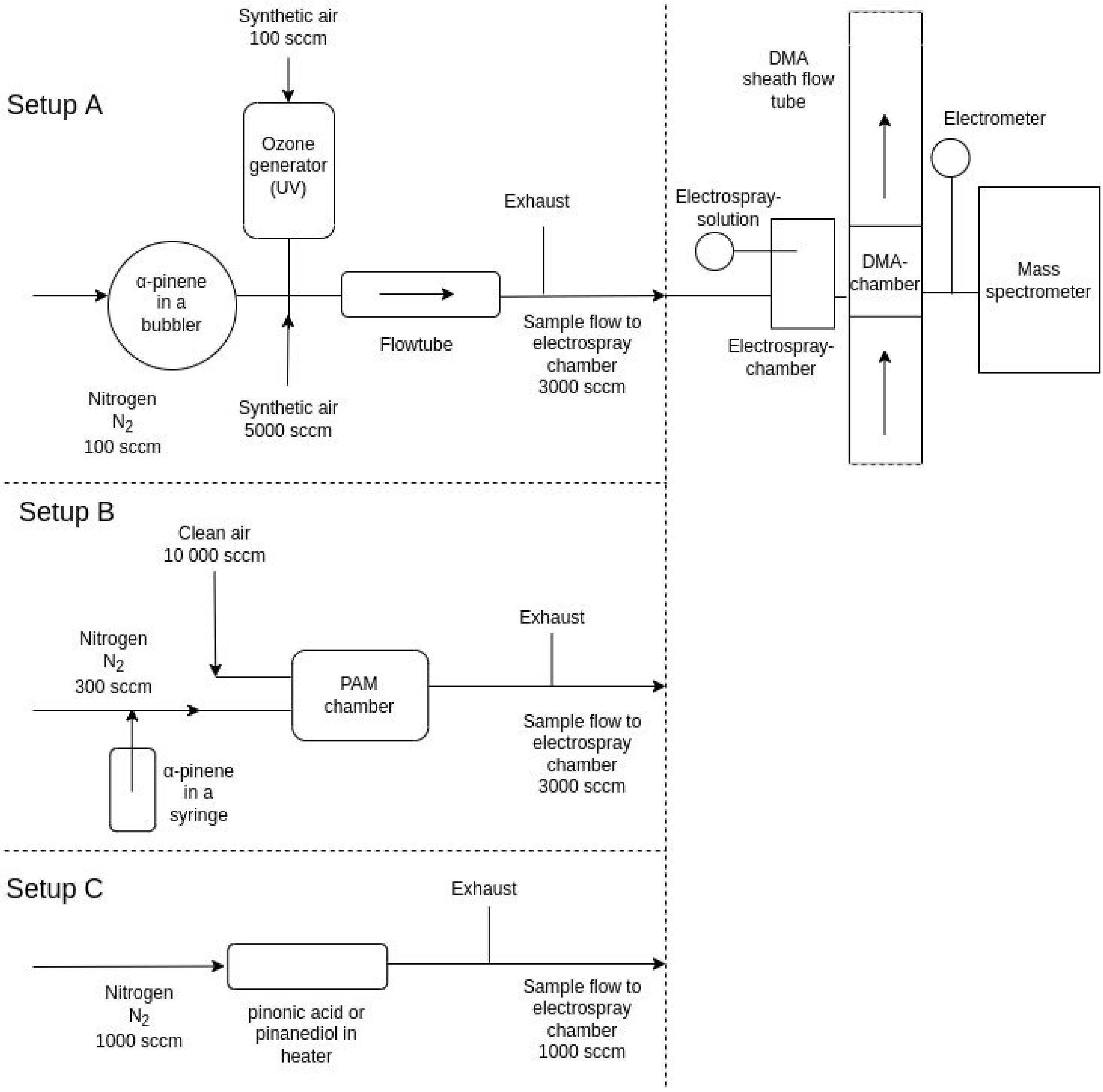

Figure S1: Experiment instrumentation with different setups to generate α-pinene ozonolysis products.

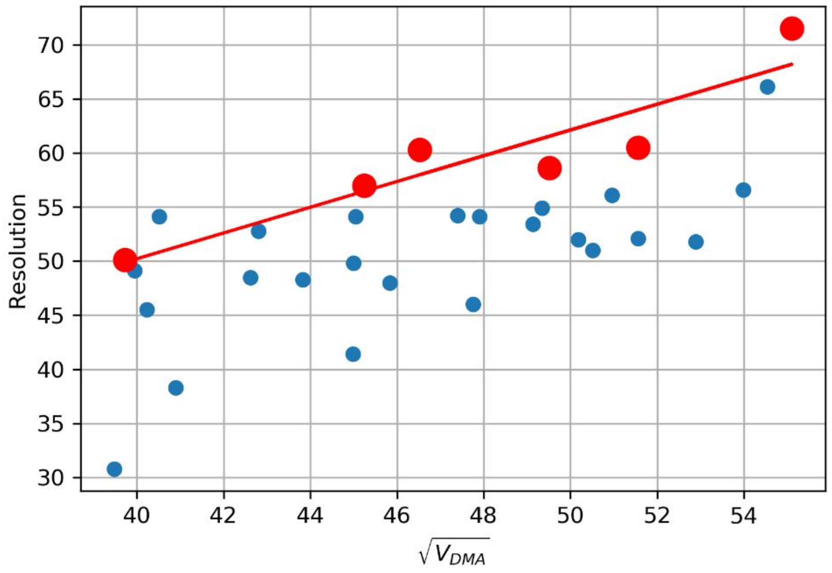

Figure S2: Mobility resolution of the DMA data (blue dots). The red dots are the peaks with the highest resolution of the DMA and the function fitted to them is used as the DMA resolution function.

Peak fitting procedures. Overlapping peaks can be separated from each other using peak fitting. For example, Fig. S3a shows the mobility spectrum of $C_{10}H_{16}O_3NO_3^-$ from PAM1 experiment. The measured spectrum clearly indicates that this peak consists of multiple isomers. Fitting three peaks with the instrument resolution obtained from Fig. S1 and calculating their sum replicates very well the measured mobility spectrum, indicating that the cluster $C_{10}H_{16}O_3NO_3^-$ has three different isomers.

Mobility peaks sometimes show peak tailing both to the left and right hand side as shown in Figure S3B and C, which complicates the peak number estimation. The number of isomers from this peak is 3+ in Table 2 in the main text. It is possible that the tail is caused by the low concentration of corresponding isomers, impurities in the sheath flow, or other imperfections in the experimental setup (Amo-Gonzalez and Fernandez de la Mora 2017). In this figure, peak tails appear on both sides of the main peaks. In the DMA, the impurities of the sheath gas can attach to the cluster of oxidation products of α-pinene and the reagent ion. These contaminated clusters have smaller mobility than the clusters without impurities, and hence they are measured at higher voltages than the original clusters. This possibly creates the right-hand side tail in the mobility spectrum (Amo-Gonzalez and Fernandez de la Mora 2017). The left-hand side tail can form due to the evaporation or restructuring of non-stable clusters (Fernandez de la Mora et al. 2020). While the origin of the peak tailing is not well known, it can be assumed that they are most likely not isomers of the measured cluster. Therefore, we do not account for the tails as isomers of the measured cluster, and only consider the highest signal of the peaks in this study.

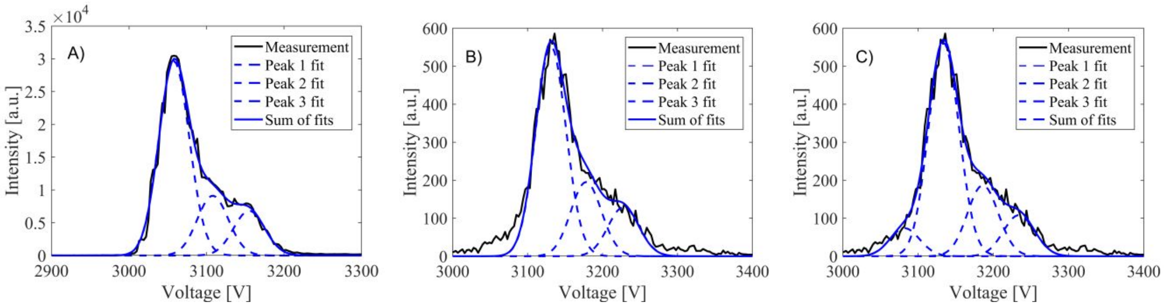

Figure S3. A) $C_{10}H_{16}O_3NO_3^-$ from PAM1 experiment. B) and C) $C_{10}H_{16}O_4NO_3^-$ from PAM1 experiment with 3 and 4 peaks fitted, respectively.

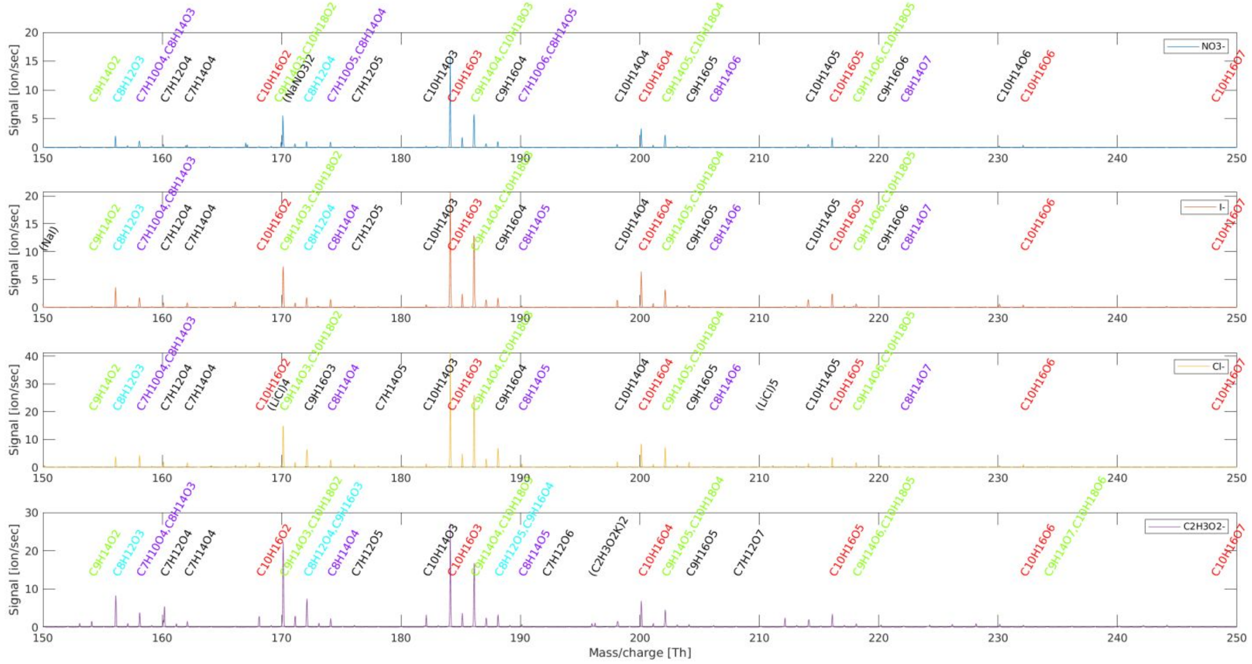

Figure S4: Mass spectrums of the measurements with negative reagent ions. Only monomers were detected.

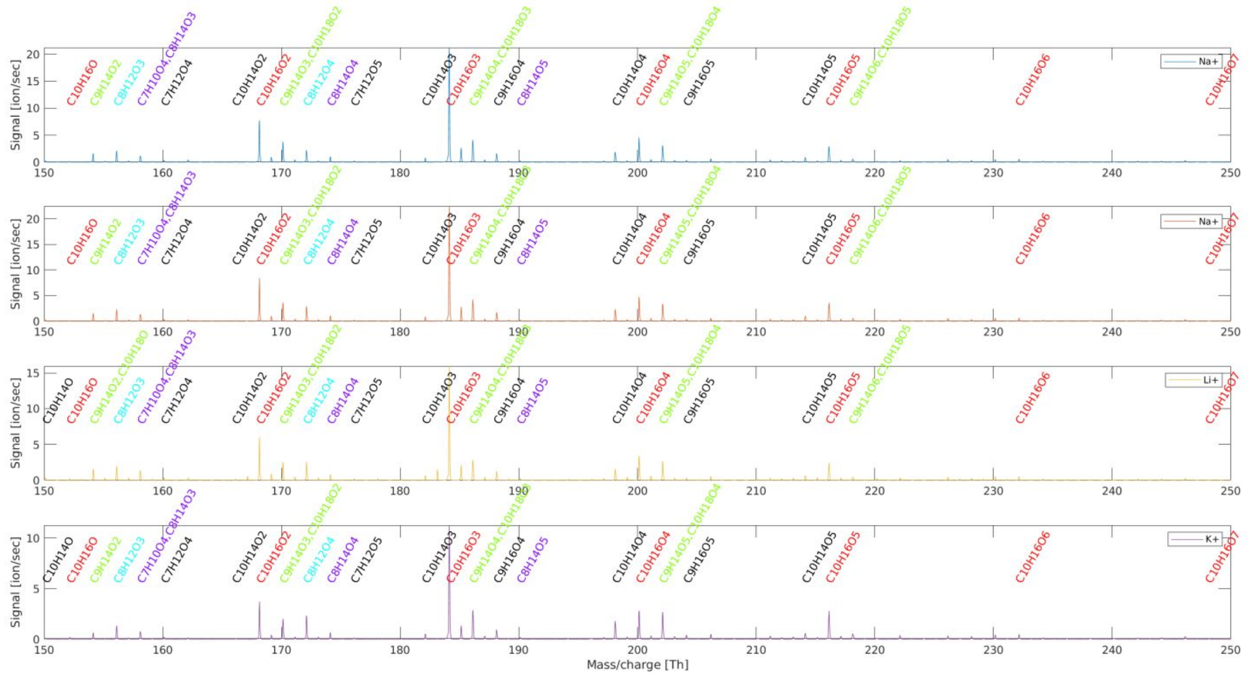

Figure S5: Mass spectrums of the measurements with positive reagent ions at the mass range of monomer clusters. The first Na+ ion is from salt NaNO3 and the second one from NaI.

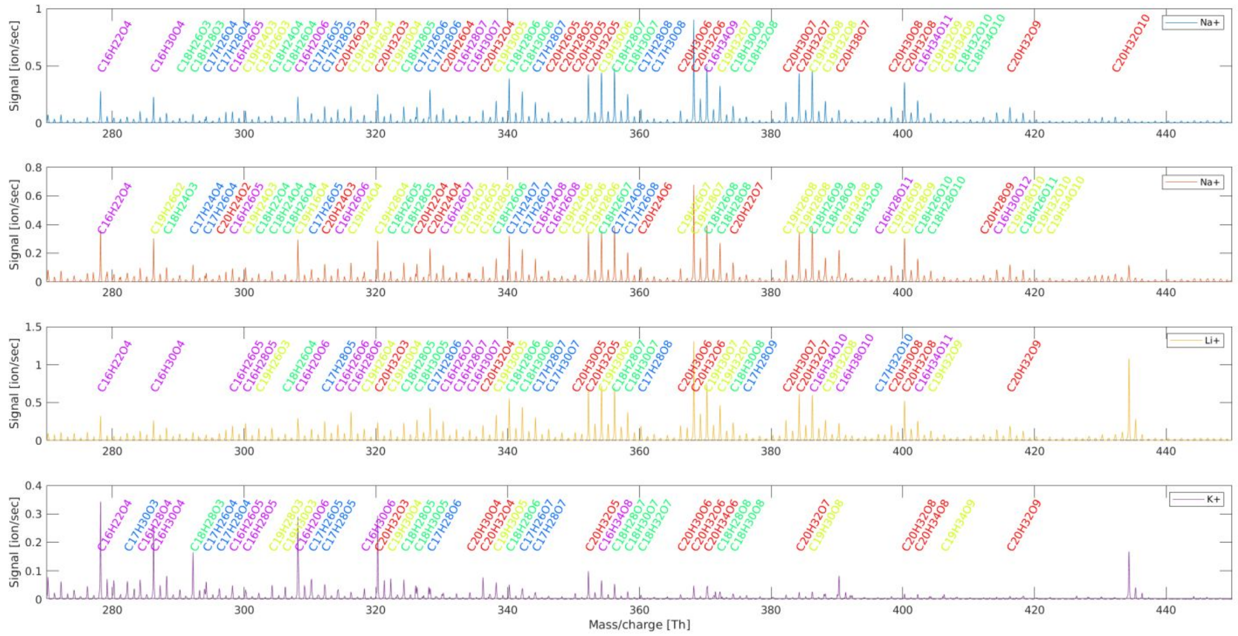

Figure S6: Mass spectrums of the measurements with positive reagent ions at the mass range of dimer clusters. The first Na+ ion is from salt NaNO3 and the second one from NaI.

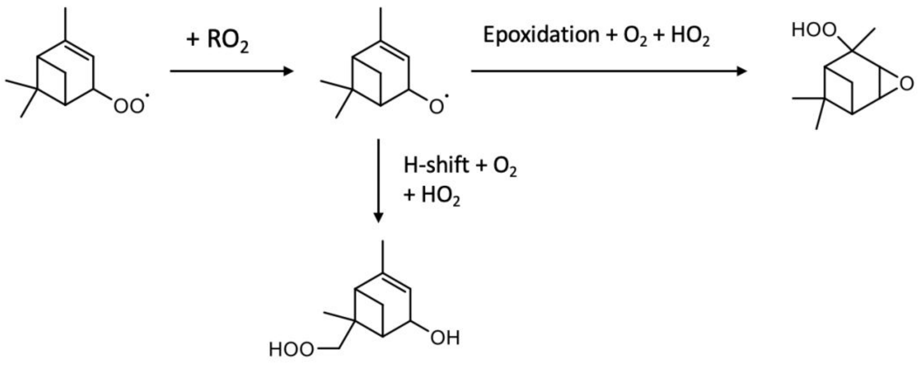

Scheme S1**:** Potential formation routes to $C_{10}H_{16}O_3$ starting from hydrogen abstraction by OH sketched according to common reaction steps.

Table S1: List of all performed experiments.

Source	a-pinene [ppb]	Ozone [ppb]	Ionization scheme	Salt	Reaget ion	Electrospray reagent [mM]
Flow tube	120 000	300	SESI	NaNO3	NO3-	0.5
Flow tube	120 000	300	SESI	NaNO3	Na+	0.5
Flow tube	120 000	300	SESI	NaI	I-	0.5
Flow tube	120 000	300	SESI	NaI	Cl-	0.5
Flow tube	120 000	300	SESI	LiCl	Li+	0.5

Flow tube	120 000	300	SESI	LiCl	C2H3O2-	0.5
Flow tube	120 000	300	SESI	C2H3O2K	K+	0.5
Flow tube	120 000	300	SESI	C2H3O2K	NO3-	0.5
PAM	31	60	SESI	NaNO3	NO3-	2.5
PAM	38	110	SESI	NaNO3	NO3-	2.5
PAM	44	350	SESI	NaNO3	NO3-	2.5
PAM	50	650	SESI	NaNO3	NO3-	2.5
Heating tube			SESI	NaNO3	NO3-	0.5-2.5
Heating tube			SESI	NaNO3	Na+	0.5-2.5
Heating tube			SESI	NaI	I-	0.5-2.5
ES			ESI	NaNO3	NO3-	1
ES			ESI	NaNO3	Na+	1
ES			ESI	NaI	I-	1

Table S2: Detected ozonolysis products charged with different reagent ions from flow tube measurements.

Compound	NO3-	I-	Cl-	C2H3O2-	Na+	Na+	Li+	K+
C7H10O4	x	x	x	x	x	x	x	x
C7H12O4	x	x	x	x	x	x	x	x
C7H12O5	x	x		x				
C7H14O4	x	x	x	x				
C7H14O5			x					
C8H12O3	x	x	x	x	x	x	x	x
C8H12O4	x	x	x		x	x	x	x
C8H12O5				x				
C8H14O3	x	x	x	x	x	x	x	x
C8H14O4	x	x	x	x	x	x	x	x
C8H14O5	x	x	x	x	x	x	x	x
C8H14O6	x	x	x					
C8H14O7	x	x	x					
C9H14O2	x	x	x	x	x	x	x	x
C9H14O3	x	x	x	x	x	x	x	x
C9H14O4	x	x	x	x	x	x	x	x
C9H14O5	x	x	x	x	x	x	x	x
C9H14O6	x	x	x	x	x	x	x	
C10H16O1					x	x	x	x
C10H16O2	x	x	x	x	x	x	x	x
C10H16O3	x	x	x	x	x	x	x	x
C10H16O4	x	x	x	x	x	x	x	x
C10H16O5	x	x	x	x	x	x	x	x
C10H16O6	x	x	x	x	x	x	x	x
C10H16O7	x	x	x	x	x	x	x	x
C10H18O2	x	x	x	x	x	x	x	x
C10H18O3	x	x	x	x	x	x	x	x
C10H18O4	x	x	x	x	x	x	x	x
C10H18O5	x	x	x	x	x	x	x	

C10H18O6				x				
C16H22O4					x	x	x	x
C16H26O5					x	x	x	x
C16H26O6						x	x	
C16H26O7						x	x	
C16H28O4								x
C16H30O4					x		x	x
C16H30O6								x
C16H30O7					x		x	
C16H30O12						x		
C16H34O8								x
C16H34O9					x			
C16H34O10							x	
C16H34O11					x		x	
C17H24O4						x		
C17H26O4						x		
C17H26O5					x	x		x
C17H26O6					x			
C17H26O7						x		x
C17H26O8						x		
C17H28O5					x		x	x
C17H28O6					x		x	x
C17H28O6					x		x	x
C17H28O8					x		x	
C17H28O9							x	
C17H30O3								x
C17H30O7							x	
C17H30O8					x			
C17H32O10							x	
C18H22O4						x		
C18H24O3						x		
C18H24O4					x			
C18H26O3					x			
C18H26O4					x	x	x	
C18H26O5					x	x	x	x
C18H26O6						x		
C18H26O8						x		
C18H26O9						x		
C18H26O10						x		
C18H26O11						x		
C18H28O3					x			x
C18H28O5					x	x	x	x
C18H28O6					x		x	x
C18H28O7					x		x	x
C18H28O8						x		x
C18H28O9						x		

C18H28O10						x		
C18H30O5							x	x
C18H30O6					x		x	
C18H30O7					x		x	x
C18H30O8					x		x	x
C18H32O7								x
C18H32O8					x			
C18H32O9						x		
C18H32O10					x			
C18H34O10					x			
C19H24O3					x	x		
C19H24O4					x	x	x	
C19H24O5						x		
C19H24O6						x		
C19H26O4					x		x	
C19H26O5						x		
C19H26O6						x		
C19H26O7						x		
C19H26O8						x		
C19H26O9						x		
C19H28O4						x		
C19H28O5						x		
C19H28O6						x		
C19H28O7						x		
C19H28O8						x		
C19H28O9						x		
C19H28O10						x		
C19H30O4					x		x	x
C19H30O5					x		x	x
C19H30O6					x			
C19H30O7							x	
C19H30O8					x			x
C19H32O7					x		x	
C19H32O8					x		x	
C19H32O9					x		x	
C19H32O10						x		
C19H34O9					x			x
C19H34O10						x		
C20H24O2						x		
C20H24O3						x		
C20H24O4						x		
C20H24O6						x		
C20H26O4					x			
C20H26O5					x			
C20H28O5					x			
C20H30O4								x

C20H30O5					x		x	
C20H30O6					x		x	x
C20H30O7					x		x	
C20H30O8					x		x	
C20H32O3					x		x	x
C20H32O4					x		x	x
C20H32O5					x		x	x
C20H32O6					x		x	x
C20H32O7					x		x	x
C20H32O8					x		x	x
C20H32O9					x		x	x
C20H32O10					x			
C20H34O6								x
C20H34O8								x

Table S3: The number of isomers of oxidation products of α-pinene from PAM chamber charged with NO_3^-.

Compound	PAM1	PAM2	PAM3	PAM4
C10H16O2	3	4-	4+	4+
C9H14O3	4	4	4	3
C10H18O2	3+	2+	3	3
C10H16O3	3	3	4-	4
C9H14O4	4	4	3+	3+
C10H18O3	3	3	4-	4
C10H16O4	3+	3+	3+	3+
C9H14O5	4	4	4+	4+
C10H18O4	3	3	3	3+
C10H16O5	4+	4+	4+	4+
C10H16O6	4+	4+	4+	4+

Table S4: Inverse reduced mobility [Vs/cm^2] and CCS [$Å^2$] of pinanediol (PD) and pinonic acid (PA) from electrospray, evaporation, flow tube and PAM experiments charged with NO_3^- , I^- and Na^+.

Source	PD NO3-	PD I-	PD Na+	PA NO3-	PA I-	PA Na+
ESI [1/Z0]	0.725	0.71	0.725	0.721	0.71	0.716
Heater [1/Z0]	0.726	0.712	0.745	0.723	0.71	0.724
Flow tube [1/Z0]	0.712	0.694	0.704	0.729	0.707	0.724
PAM [1/Z0]	0.716	-	-	0.726	-	-
ESI [CCS]	156	150	157	154	150	155
Heater [CCS]	156	151	161	155	150	156
Flow tube [CCS]	153	147	153	156	150	156
PAM [CCS]	154	-	-	155	-	-

Table S5: Inverse reduced mobility [Vs/cm²] for the analyzed oxidation products of α-pinene clustered with different reagent ions from PAM and flow tube experiments. The first Na^+ ion is from salt $NaNO_3$ and the second one from salt NaI.

Compound	PAM NO3-	NO3-	I-	Cl-	C2H3O2-	Na+	Na+	Li+	K+
C10H16O2	0.719	0.726	0.696	0.709	0.754	0.706	0.707	0.707	0.708
C9H14O3/C10H18O2	0.716	0.712	0.694	0.708	0.748	0.704	0.705	0.705	0.7
C10H16O3	0.726	0.729	0.707	0.709	0.747	0.724	0.725	0.724	0.72
C9H14O4/C10H18O3	0.729	0.734	0.707	0.715	0.749	0.722	0.725	0.728	0.72
C10H16O4	0.741	0.744	0.722	0.732	0.761	0.743	0.744	0.742	0.738
C9H14O5/C10H18O4	0.742	0.742	0.72	0.729	0.763	0.741	0.74	0.731	0.733
C10H16O5	0.751	0.749	0.733	0.734	0.761	0.762	0.764	0.758	0.757
C10H16O6	0.764	0.752	0.746	0.74	0.768	0.774	0.776	0.773	0.764

Table S6: Collision cross section [Å²] for the analyzed oxidation products of α-pinene clustered with different reagent ions from PAM and flow tube experiments. The first Na+ ion is from salt NaNO3 and the second one from salt NaI.

Compund	PAM NO3-	NO3-	I-	Cl-	C2H3O2-	Na+	Na+	Li+	K+
C10H16O2	154	156	147	153	162	153	153	154	153
C9H14O3/C10H18O2	154	153	147	153	160	153	153	154	151
C10H16O3	155	156	150	152	160	156	156	157	155
C9H14O4/C10H18O3	156	157	150	154	160	156	157	158	155
C10H16O4	158	158	152	157	162	160	160	160	158
C9H14O5/C10H18O4	158	158	152	156	163	159	159	158	157
C10H16O5	160	159	154	157	162	163	164	163	162
C10H16O6	162	159	157	158	163	165	166	166	163

References

Amo-Gonzalez, M. and Fernandez de la Mora, J. (2017). Mobility Peak Tailing Reduction in a Differential Mobility Analyzer (DMA) Coupled with a Mass Spectrometer and Several Ionization Sources. J Am Soc Mass Spectr 28:1506-1517.

Fernandez de la Mora, J., Genoni, M., Perez-Lorenzo, L. J., Cezairli, M. (2020). Measuring the Kinetics of Neutral Pair Evaporation from Cluster Ions of Ionic Liquid in the Drift Region of a Differential Mobility Analyzer. J Phys Chem A 124:2483-2496.

Gabelica, V., Shvartsburg, A. A., Afonso, C., Barran, P., Benesch, J. L. P., Bleiholder, C., Bowers, M. T., Bilbao, A., Bush, M. F., Campbell, J. L., Campuzano, I. D. G., Causon, T., Clowers, B. H., Creaser, C. S., De Pauw, E., Far, J., Fernandez-Lima, F., Fjeldsted, J. C., Giles, K., Groessl, M., Hogan, C. J., Jr., Hann, S., Kim, H. I., Kurulugama, R. T., May, J. C., McLean, J. A., Pagel, K., Richardson, K., Ridgeway, M. E., Rosu, F., Sobott, F., Thalassinos, K., Valentine, S. J., Wyttenbach, T. (2019). Recommendations for reporting ion mobility Mass Spectrometry measurements. Mass Spectrom Rev 38:291-320.

Ude, S. and Fernandez de la Mora, J. (2005). Molecular monodisperse mobility and mass standards from electrosprays of tetra-alkyl ammonium halides. J Aerosol Sci 36:1224-1237.
